# Effects of Zinc Sulfate or Propylene Glycol on Intake, Digestibility, and Forage Selection by Grazing Sheep in a Semi-Arid Region During the Rainy Season

**DOI:** 10.3390/ani9110867

**Published:** 2019-10-25

**Authors:** Hélio Costa, Eloisa Saliba, Diego Galvani, Marco Bomfim, Ângela Maria Lana, Ana Luiza Borges, Aline Landim, Antonio Faciola

**Affiliations:** 1 Department of Animal Science, Federal University of Minas Gerais, Belo Horizonte 123-970, Brazil; saliba@ufmg.br (E.S.); lana@vet.ufmg.br (Â.M.L.); analuizavetufmg@gmail.com (A.L.B.); 2 Embrapa Goats and Sheep, Estrada Sobral-Groaíras, Sobral 62010-970, Brazil; diego.galvani@embrapa.br (D.G.); marco.bomfim@embrapa.br (M.B.); 3 Department of Animal Science, Vale do Acaraú State University, Sobral, CE 62040-370, Brazil; alinelandim@yahoo.com.br; 4 Department of Animal Sciences, University of Florida, Gainesville, FL 32611, USA; afaciola@ufl.edu

**Keywords:** *Caatinga*, native pasture, forage quality, ruminal extrusa sample, selectivity, tannins

## Abstract

**Simple Summary:**

*Caatinga* is an important biome in Brazil’s semi-arid region, it is characterized by low precipitation and great plant availability seasonality. In this context, mineral and energy supplementation may improve pasture consumption and nutrient utilization. The objective of this study was to determine intake, nutrient availability, and animal selection of major forage species by sheep supplemented with zinc sulfate or propylene glycol in *Caatinga*-native pastures during the rainy season. Twenty-four sheep were distributed into three treatments (control, Zn, and propylene glycol supplement) in this 112-day study. There was no effect of treatments on plant selection. However, plant species selected by sheep changed over time. Generally, greater intakes were found in April compared to May. In conclusions, based on the finding of this study, Zn and PG supplementation did not improve sheep nutrient intake when grazing *Caatinga*-native pasture in the rainy season.

**Abstract:**

The objective of this study was to determine intake, nutrient availability, and animal selection of major forage species in sheep supplemented with zinc sulfate or propylene glycol in *Caatinga*-native pastures during the rainy season. Twenty-four mixed Santa Inês sheep, all non-castrated males, with initial weight of 19.3 ± 2.52 kg and 4 ± 0.35 months of age, were distributed in a complete randomized design into three treatments: Control (CT)—concentrate supplemented at 0.7% of body weight; CT + 300 mg of Zn day^−1^; CT + 2.5 mL of propylene glycol/kg LW^0.75^·day^−1^. Measurements were done in four periods during the rainy season, with 28 days of interval between each measurement. Differences were observed in the composition of the ruminal extrusa samples from pastures for crude protein (CP) (192 to 131 g kg^−1^), *in vitro* dry matter digestibility (IVDMD) (537 to 441 g kg^−1^), and *in vitro* organic matter digestibility (IVOMD) (468 to 359 g kg^−1^) in March and June, respectively. There was no effect for treatments, neither for the treatment x period interaction on organic matter (OM), CP, and fibrous fraction intake (*p* > 0.05). Organic matter intake (OMI) was, on average 23.9% greater in March compared to June. CP intake decreased monthly (*p* < 0.05). Fibrous fraction intake was greater in March (*p* < 0.05), with reductions of 34.8, 33.3, and 39.4% in June, respectively, for neutral detergent fiber (NDF), acid detergent fiber (ADF), and cellulose (CEL) fractions. There was no effect of treatments (*p* > 0.05) on selection of vegetal species present in the pasture. On the other hand, the proportion between ingested species changed over the experimental period. Greater intakes were found in April compared to May, except for *Zizyphus joazeiro* intake, which was greater in March (*p* < 0.05). In conclusion, based on the finding of this study, Zn and propylene glycol (PG) supplementation did not improve sheep nutrient intake when grazing *Caatinga*-native pasture in the rainy season. *Caatinga*-native pasture biomass has adequate protein and digestible organic matter levels during early rainy season. Over this period, however, the advanced maturity of the plants and the reduced availability of pasture may result in variations of intake by the animals. In the months of April to June, a reduced energy supply is caused by reduced nutritive values of pastures, which contributes to inefficient protein utilization and reduced feed intake.

## 1. Introduction

*Caatinga* is the predominant vegetation of an important biome in the semi-arid region in Northwestern Brazil. It is characterized by a diversity of forage species in different strata (herbaceous, shrub, and arboreal), with approximately 70% of species in the diet of ruminants [1,2,3]. Food availability for production of small ruminants in the Brazilian semi-arid region is marked by the seasonality of forage production throughout the year. Because rains are concentrated in a short period (February to June), forage availability and quality are compromised when grazing is extended to the dry season. These aspects negatively affect production and, therefore, understanding the utilization of these species during the period of greater availability may optimize nutrient utilization and allow for greater production.

Supplementation strategies may improve the efficiency of pasture utilization. Some minerals have important functions in the ruminal environment, contributing to maintaining osmotic pressure, buffering capacity, and dilution rate. Zn supplementation in the form of ZnSO_4,_ for instance, coupled with protein supplementation, is capable of increasing dry matter (DM) digestibility of forages [4]. This strategy can be particularly interesting for animals kept in native pastures in the *Caatinga* biome, since it has low available energy but adequate CP levels [5,6].

Propylene glycol (PG) has been used to increase energy supply and as glycogen precursor in ruminants. It is metabolized by ruminal microbiota and reduced to n-propanol or lactate, being initially converted into propionaldehyde by dehydration processes. PG supply increases the concentration of propionate in the rumen, indirectly through lactate formation; then, it can be converted to glucose in the liver [7]. PG fermentation in the rumen is also characterized by inhibiting methane production, resulting in lower energy loss [8].

The hypothesis of this study was that zinc sulfate and propylene glycol would improve intake and nutrient utilization in *Caatinga*-native pastures in the rainy season. The objective of this study was to determine intake, nutrient availability, and animal selection of major forage species in sheep supplemented with zinc sulfate or propylene glycol in *Caatinga*-native pastures during the rainy season.

## 2. Materials and Methods

### 2.1. Ethics and Animal Usage

All procedures involving animals were carried out in accordance with protocols approved by the Ethics Committee on Animal Usage from the Federal University of Minas Gerais (CEUA/UFMG, No 321/2013).

### 2.2. Location and Characterization of Experimental Area

**Figure 1:**
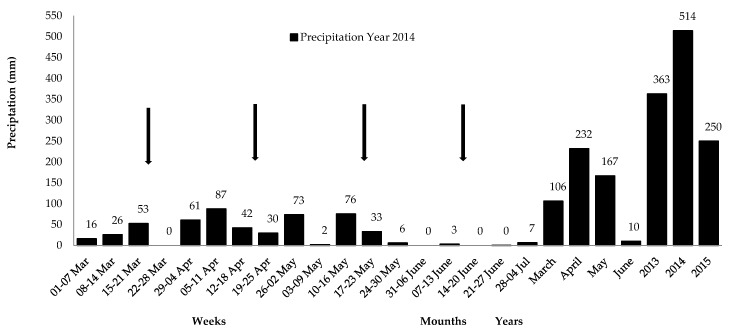
Weekly and monthly precipitation from March to June 2014, and yearly precipitation during the assessment, in 2013, 2014, and 2015. Arrows indicate collection weeks in each month. Source: [10].

The research was conducted at Embrapa Goats and Sheep, Sobral, Ceará, in the northeast of Brazil (3°45’51.59” S and 40°21’04.24” O, 92 m above sea level (a.s.l.). It used eight hectares of a *Caatinga*-native pasture area managed according to Silva [9]. The area’s predominant soils were litolic dystrophic, planosol, and non-calcium brown. The experiments were executed during the rainy season (March–June 2014), with precipitation of 514 mm (Figure 1), and average temperature and air humidity of 26.5 °C and 78.0%, respectively [10].

### 2.3. Treatments and Experimental Animals

Twenty-four sheep were used: crossbred, non-castrated Santa Inês males, with initial body weight of 19.3 ± 2.52 kg, four months of age, eight animals per treatments, distributed in a complete randomized design into three treatments: Control (CT)—concentrate supplementation at 0.7% of body weight; CT + 300 mg of Zn day^−1^ in the form of heptahydrate zinc sulfate (ZnSO_4_.7H_2_O); CT + 2.5 mL propylene glycol/kgLW^0.75^ day^−1^.

Animals were kept in continuous stocking and weighed weekly to monitor daily weight gain and supplementation feeding. All animals had free access to mineral salt, whose composition was: Ca = 82.0 g/kg, Co = 30.0 mg/kg, Cu = 350 mg/kg, Cr = 11.7 mg/kg, S = 11.7 g/kg, P = 60.0 g/kg, I = 50.0 mg/kg, Mn = 1200 mg/kg, Mo = 180 mg/kg, Se = 15 mg/kg, Na = 132 g/kg and Zn = 2600 mg/kg.

The daily Zn dosage required to increase concentration in ruminal fluid at 300 mg Zn day^−1^ was calculated. The amount of Zn was established considering the concentration in the mineral salt, and the complementary addition of ZnSO_4_.7H_2_O. The amount of salt and zinc sulfate supplied to the animals were weighed and mixed prior to being provided and adjusted so as to not contain leftovers. For Zn supplementation, the procedures described by [11] and the maximum tolerable level of toxicity for sheep according to NRC (2007) [12] were used. PG was supplied at 2.5 mL × kgLW^0.75^ animal^–1^ day^–1^ [7] and mixed directly into the concentrate. PG supply was adjusted weekly, according to the group’s average weight in kgLW^0.75^ (n = 8).

A stocking rate of 0.4 ha head^−1^ was used, considering an animal of 30 kg of LW [1]. The animals were taken to the pasture at 07:00 and brought back at 16:00, when they were supplemented according to treatments. The concentrate was composed of corn (540 g kg^−1^ DM), soybean meal (451 g kg^−1^ DM) and limestone (9.0 g kg^−1^ DM), formulated as recommended by the NRC (2007) [12], for finishing category with predicted average daily gain of 150 g.

### 2.4. Botanical Composition and Forage Availability

Before the start of the experimental period, the occurrence of the main forage groups and species were determined using the method proposed by Araújo Filho [1], with a frame measuring 0.25 m^2^ and systematically arranged along lines, every 4 m, totaling 50 sampling points. The percentage of the main forage species of the herbaceous stratum in the area was analyzed. Forage availability in weight was estimated by collecting the forage from the herbaceous stratum contained within the frame every 12 m. The material was weighed and oven dried at 55 °C for 72 h to calculate DM ha^–1^ availability.

### 2.5. Nutrient Intake, Digestibility, and Forage Selection

Four intake and digestibility trials were carried out in the rainy season during the months of March to June, with an interval of 28 days between periods. Total nutrient intake and intake partition were determined, identifying the main forage plants contained in the grazing area and potentially ingested by the animals. To determine total intake, external indicator LIPE^®^ (patent No BR0304736-9) was administered orally in the morning, at a dose of 0.25 g per animal day^–1^, for a period of seven days, with two days for adaptation and stabilization of the indicator in the gastrointestinal tract, and five days for fecal collection [13]. Fecal samples were collected directly from the animals’ rectum, stored in plastic bags and frozen in a freezer at −20 °C. Samples composed by animal, by period, were dried at 55.0 °C for 72 h and milled so as to determine LIPE^®^ concentration in the feces and estimate fecal production (FP), as per the equation below:

Fecal production, g day^−1^ = (LIPE^®^ supplied, g/LIPE^®^ recovered in feces, g) × fecal DM, g kg^−1^ (1)

To assess digestibility and forage chemical composition, two adult sheep were used, with mean weight of 34.5 ± 2.1 kg, cannulated in the rumen. Ruminal extrusa samples were collected as described by Olson [14], for five consecutive days in each experimental period, starting one day before feces collection in the animals used for intake determination.

Total OM intake was calculated using fecal DM production estimated by a LIPE^®^ indicator [15]:

Intake (g OM day^−1^) = Production of fecal DM (g day^−1^)/1−IVOMD/100 (2)

where IVOMD is the apparent OM digestibility determined according to [16]. To correct for mineral contamination via saliva, in the ruminal extrusa sample intake was corrected for OM [17].

In order to establish intake partition by sheep and determine the main species ingested, a double marker procedure was used (LIPE^®^, external; Klason Lignin—KL, internal), adapting the *n-alkanes* formula used by Dove and Meyes (1991), according to Silva [18]. The use of key species was adopted as criterion for insertion in the model used in order to quantify intake partition. Key species consisted of a typical set of species present in an ecosystem, providing a structure that suits the floristic community of the area, constituting the largest possible number of native species. For selection of key species, information about the main herbaceous species were considered, obtaining frequencies, selecting those with greater distribution in the area and forage potential. A survey of phytosociological studies and research that have determined the botanical composition of the selected diet for sheep in *Caatinga*-native pasture areas [2,6,19,20,21,22,23,24,25,26] was carried out also as a criterion to select the main key species. A total of 19 species were selected, classified as grasses (n = 3) and legumes (n = 13), both from the herbaceous stratum, and arboreal species (n = 3), according to their incidence in the area.

The species’ intake partition was determined by considering the amount of LIPE^®^ supplied, suggested as an external indicator, and its concentration in the feces. KL concentration in the different potential forage species selected in the experimental area was considered, as well its concentration in the feces, with KL being suggested as internal marker, by adapting the alkane formula described by Dove [27], as recommended by Silva [18], as follows:

Intake (g DM day^−1^ kg^−1^) = LIPE^®^ supp, g day^−1^ kg^−1^/[(LIPE^®^ fec, g day^−1^ kg^−1^)/(LK fec, g day^−1^ kg^−1^−LK supp, g day^−1^ kg^−1^)] × LK supp, g day^−1^ kg^−1^ (3)

where LIPE^®^ supp is the amount of LIPE® provided to the animals−0.25 g animal^–1^ day^–1^; LIPE^®^ fec is the amount of LIPE^®^ recovered from the feces of each animal^−1^ day^−1^; KL fec is the Klason lignin determined in each fecal sample; and KL forage is the Klason lignin determined in each forage sample.

To determine digestibility coefficients (%), intake and fecal production data were used. Nutrient digestibility was calculated according to Lascano [28].

### 2.6. Chemical Analyses

Ruminal extrusa, fecal, and forage samples were dried at 55 °C for 72 h, and, together with the concentrate, milled in a knife mill with 1 mm sieves. They were analyzed for DM (method 934.01), ash (method: 938.08), CP (method 968.06) in Leco^®^ equipment (CN628, St Josesh, MI, USA), and ether extract (EE) (method 920.39) according to the Association of Official Analytical Chemists International (AOAC 1990) [29]. OM was calculated as the difference between DM and ash content aNDFom-NDF and ADF were analyzed according to Goering [30], with adaptation for autoclave analysis according to Senger [31]. Acid detergent lignin (ADL) content was analyzed (method 973.18D) according to AOAC (1990) [29], neutral detergent insoluble nitrogen according to Licitra [32], and KL was analyzed by acid hydrolysis [33]. Total tannins were analyzed using the Folin–Ciocalteu method [34].

### 2.7. Statistical Analysis

To assess parameters of nutrient intake, intake selection and digestibility, a complete randomized design was used. Effects of treatment and periods were determined using the following statistical model:

Y_ijkl_ = μ + T_i_ + a_ij_+ P_k_ + (T × P)_ik_ + e_ijkl_ (4)

where μ = overall mean; T_i_ = fixed effect of treatments (i = CT; Zn; PG); a_ij_ = random residual effect associated with the animal; P_k_ = fixed period effect (k = March; April; May; June); (T × P)_ik_ = treatment × period interaction; and e_ijkl_ = experimental error associated with the observation of the animal each month. Means were compared by the Tukey–Kramer test, with a significance of 0.05. The Proc GLM procedure of the Statistical Analysis System 9.0 (SAS Inst. Inc., Cary, NC, USA) was used. A descriptive analysis was performed to assess the availability and frequency of the herbaceous species.

## 3. Results

The herbaceous stratum was composed of 28% of species from legumes and 72% of species from grasses, predominantly from *Euphorbiaceae, Poaceae*, and *Fabaceae* families. The pasture area at the beginning of the study showed availability of total DM from the herbaceous stratum of 1897 kg hectare^–1^, favored by adequate rainfall average in the first months of the rainy season (Table 1; Figure 1).

The most frequent herbaceous species in the area were *Arachis dardani* Krapov., & W.C. Greg., *Oxalis corniculata* L., *Hyptis suaveolens*, *Alternanthera tenella colla*, *Acalypha communis*, *Aspilia martii* Baker, *Centrosema Pascuorum* Mart. Ex Benth., *Alternanthera brasiliana* (L.) Kuntze), *Stylosanthes humilis*, *Merremia aegyptia*, *Commelina diffusa*, *Digitaria Sanguinalis* (L.). Scop, *Wissadula rostrata*, in which CP levels of legumes ranged from 125 to 295 g kg^−1^ of CP (Table 2 and Table 3).

To better characterize the pasture, the dietary nutrient composition of the ruminal extrusa samples were determined in different months of the experimental period (Table 4). Variations were evidenced as to the composition of the ruminal extrusa sample over the months for CP (192–131 g kg^−1^), DM digestibility (537–441 g kg^−1^) and OM digestibility (468 and 359 g kg^−1^) in the months of March and June, respectively (Table 4).

There was no effect for treatments and treatment x period interaction as to OM intake (OMI), CP intake (CPI), and fibrous fractions NDF intake (NDFI), ADF intake (ADFI), Cellulose intake (CELI) in g day^−1^ and g/kgLW^0.75^ (*p* > 0.05; Table 5).

For OMI in the different periods, greater intakes were obtained at the beginning of the rainy season (March) compared to the other months. OMI was 23.9% greater in March compared to June. For CPI, there was a monthly reduction in intake, with a greater difference between the first and the last months of the studied period, being 54.5% lower in June compared to March (*p* < 0.05; Table 5).

The intake of fibrous fractions over the periods was greater in March (*p* < 0.05; Table 5). In June, intake was lower than in the initial period (March), with a reduction in NDFI (34.8%), ADFI (33.3%), and CELI (39.4%). At the same time, there was a pattern of intake reduction for the other periods (April and May) in relation to the initial period, which was, however, more evident in the month of June. A greater organic matter digestibility (OMD) was found for PG. Concerning ADFD, there were greater coefficients for CT and PG (*p* < 0.05; Table 6). Considering the digestibility coefficients in the periods, values were greater in March, at the same time, with a reduction in the digestibility of CP (57.0%) and fibrous fractions (NDF, 39.7%; ADF, 36.4%; CEL, 46.5%) in June (*p* < 0.05; Table 6).

The intake partition assessment took into account key species, recognized with greater contribution to sheep’s diet during the rainy season. The main key species established were subdivided into grasses: *Cyperus uniciualatus* Schrad. ex Ness, *Cynodon* sp., *Digitaria Sanguinalis* (L.). Scop; herbaceous legumes: *Alternanthera brasiliana* (L.) Kuntze, *Alternathera tenella* Colla, *Amaranthus blitum*, *Arachis dardani*, *Aspilia martii* Baker, *Borreria verticillata*, *Centrosema pascuorum* Mart. Ex Benth., *Commelina diffusa*, *Delilia biflora* (L.) Kuntze, *Oxalis corniculata* L., *Sesuvium portulacastrum*, *Stylosanthes humilis*, *Wissadula rostrata*); and some arboreal legumes species: *Auxemma oncocalix*, *Mimosa caesalpinifolia and Zizyphus joazeiro*, totaling 19 species (Table 7).

For intake partition, there was no effect of treatments (*p* > 0.05) for species selection. Over the periods, there were no major changes in the proportion of species ingested during the experiment (*p* < 0.05; Table 7). Overall, the results indicate that the ingestion for each species was variable with the months, but between species the behavior was similar. Although floristic composition in each month was not determined, there seems to be, by comparison among species, little variation in intake by the animals. Greater intakes were observed in April compared to May, except for *Zizyphus joazeiro* intake, which was greater in March (*p* < 0.05; Table 7).

## 4. Discussion

Evaluating goats supplemented in the finishing phase on *Caatinga*-native pasture, [35] observed correlations in DM and CP concentration during the rainy season, in May and June, and, at the end, in July, leading to a reduction in the levels of these fractions. The values obtained in the current research seem adequate to meet the protein requirements of animals raised on this type of pasture, emphasizing that one should observe how much of this protein is degradable in the rumen for utilization by microorganisms.

Pfister and Malechek [3] reported a gradual decline in CP levels of diets selected by sheep and goats in the rainy season from 180 g kg^−1^ of CP in May to 120 g kg^−1^ of CP in December in *Caatinga* pasture. Still, in said study, at the beginning of the rainy season, CP levels were close to 250 g kg^−1^, then dropped (170 g kg^−1^ CP) in the middle of the rainy season (April).

Free-grazing ruminants are selective as to what to eat and, in general, select the diet with better quality, i.e., greater digestibility and protein levels, and fewer secondary compounds than the average vegetal biomass supplied [12]. Thus, even in the rainy season with a greater supply of forage mass constituted by varied amount of species, changes naturally occur in the proportion and quality of dietary constituents consumed by grazing sheep over this period.

The NRC (2007) [12] establishes for live-weight lambs similar to those of the current study an intake of 64.5 g of DM/kgLW^0.75^. Considering the average OM value (810 g kg^−1^) of the selected diet in the different periods, and that, from this total, the OM requirement would be 52.2 g/kgLW^0.75^, a deficit was verified in this intake only at the end of the rainy season (June). For CPI, the recommendation is 11.7 g CP/kgLW^0.75^. Not considering any supplementation, and as verified by the CPI and CP percent in ruminal extrusa samples observed in the current study, CP requirements were only met in March.

Lower ingestion in the final periods is related to changes in grazing behavior, affected by the greater rainfall concentration in April and May and to the pasture’s lower nutritional availability and quality in June, notably IVOMD (Figure 1; Table 4). In addition, changes in intake are caused by grazing pressure, since the animals remained in the area during the entire finishing phase, but also by a lower availability of species that the animals prefer, and the poor quality of the diet ingested [26], according to our study in June compared to March.

In a study evaluating the effect of grain supplementation on intake and digestibility of pasture and diets by goats in *Caatinga* pasture, in the rainy season, total OMI was observed for non-supplemented and supplemented animals (0.6% LW, value close to that of our study) of 325 and 377 g day^−1^, and 50.5 and 54.4 g/kgLW^0.75^, respectively [2].

Studies have indicated that CP intake by sheep in *Caatinga*-native pasture during the rainy season was not a limiting factor to meet the animals’ demand [24,25]. However, this aspect only considers the pasture’s crude protein levels, and it is important to quantify how much of this fraction is available for utilization by microorganisms. This fact indicates that the utilization of protein fractions correlates with the availability, or even with factors associated with these proteins, such as secondary compounds, such as total tannins and lignin, which affect the degradation of protein constituents in the rumen [34]. This condition, if not taken into account, impairs the indication of an adequate utilization of *Caatinga* pasture, showing the need for strategic supplementations to adjust nutrient intake and maximize performance.

Some of the species that were collected in the first two months of the current study had adequate CP levels, with *Digitaria Sanguinalis* (L.). Scop standing out in the grass group, and *Alternathera tenella* Colla, *Commelina difusa*, *Borreria verticillata*, *Stylosanthes humilis*, *Wissadula rostrata*, and *Oxalis corniculata* L. standing out among the legumes, but also with elevated lignin levels (Table 4). During the rainy season months there are changes in the chemical composition of plants, with increased formation of CP-lignin bonds, which may lead to inefficient utilization of the diet’s CP by the animal (in the rumen) due to reduced digestibility over these months (Table 4).

Changes in the botanical composition of the diet in this period derive from a lower availability and quality of herbaceous fractions, as well as edible portions of shrubs and trees. However, part of the diet at the end of the rainy season can be composed of leaves of deciduous species, due to a decline in available biomass [3]. Although usually not considered as part of forage in traditional plant inventories for grazing use, litterfall, made up by leaves of deciduous trees (such as *Auxemma oncocalix*, *Mimosa caesalpinifolia*, *Zizyphus joazeiro*), can be an important dietary component of grazing sheep, mainly at the end of the rainy season, extending to the dry season [5]. Probably, these conditions and the greater presence of total tannins (14.8 g kg^−1^) at the end of the rainy season, in June (Table 4), favored reductions in OMI and CPI, and their consequent digestibility, as well as in fibrous fractions.

This research found no major changes in NDF levels obtained from the pasture during the periods, with a mean value of 637 g kg^−1^, but there was a reduction in IVOMD by 23.3% when the rain began, compared to April, May, and June, resulting in lower intake during these periods. Intake is inversely related to NDF levels in diets with protein values of 60–80 g kg^−1^ and NDF greater than 600 g kg^−1^ [36,37], since there is correlation between NDF and volume and/or energy density of foods. On the other hand, intake by grazing animals is also influenced by the digestibility of the diet ingested [36]. The supply of zinc sulfate and propylene glycol did not increase the intake and the efficiency of use of dietary nutrients in pasture. Greater OM and ADF digestibility for PG when compared to Zn may be favored by the low energy input from PG to microorganisms, providing conditions to improve fiber utilization in the rumen.

Based in our findings, the greatest intakes of dietary nutrients in the rainy season in the native pasture of the semi-arid region are a consequence of the greater amount and quality of pastures in the beginning and middle of the rainy season. Due to the ephemerality of some species, there are changes in the constitution of pasture canopies and succession by other species. Native pasture areas with adequate stocking rates allow for intake selectivity, making it possible to meet the requirements of sheep, especially as to CP, with emphasis on the availability aspects of this fraction.

The interaction of some factors may explain differences in intake and digestibility among months in the rainy season. First, there was probably greater selective activity by the animals, with most of the dietary composition containing a blend of herbaceous legumes and fragments of shrub leaves, mainly from May to June, resulting in lower IVOMD. The digestibility of these constituents is moderately low [3]. Second, the secondary polyphenolic compounds in these species, such as tannins, favored reductions in IVOMD and CPD and ADFD [3].

Moreover, during the collection periods, even without botanical quantification, a greater participation of fractions of shrub and tree leaves, and of dry *Auxemma oncocalix* and *Mimosa caesalpinifolia* leaves were observed as of May. These species had greater lignification with levels at 234 g kg^−1^ (*Auxemma oncocalix*) and 212 g kg^−1^ (*Mimosa caesalpinifolia*) of lignin, favoring a decline in protein and digestibility levels. This reduction is attributed to the greater participation of stem and leaves of wood plants rich in secondary compounds, which, in turn, increases with the greater participation of shrubs in the diet at the end of the rainy season [24].

The large contribution of annual plants such as herbaceous legumes occurs due to the greater distribution of the species in the area and, at the same time, they present a longer phenological cycle compared to grasses, which are more ephemeral. Grasses and herbaceous legumes account for about 70% of ruminants’ diet in the rainy season, and, specifically, in sheep’ diet, the participation of grasses and broadleaf herbs constitutes 85.9% [1]. Considering the participation of each plant in the diet composition regarding intake partition by sheep in the four periods, and relating it to chemical composition (e.g., CP and NDF), herbaceous legume species contributed, on average, 73.4% of CPI and 61.7% of total NDFI in the ingested diet. This aspect indicates that when there is availability of herbaceous stratum composed of a larger fraction of legumes, resulting in a greater condition to meet requirements, e.g., CP, due to the high levels of this nutrient that are contained in these species (125–295 g kg^−1^ of CP). As for concepts of availability and quality in native pasture for pastoral purposes, protein does not seem to be the limiting nutrient, especially in the rainy season, if these aspects are met.

In the current study, even with the adequate protein concentration in the species, CPI levels stood below 11.7 g/kgLW^0.75^ during the months of April, May, and June [12]. In addition, the low digestibility values during these months also affected CPI. Probably the increase of total tannins content in the selected diet during these months may have contributed for these reduction. In semiarid regions native pastures CP does not seem to be a limiting factor, but rather, the energy concentration, which negatively affects nutrient intake and utilization [38].

The composition of the diet obtained was influenced by the month of collection, since there is variation in dietary composition, which is directly related to forage availability throughout the year and regulated by rainfall, which leads to the full development of plants in different seasons, being greater in the rainy season [6]. In this period, sheep and goats selected diets containing herbaceous legumes, sprouts and leaves of trees and shrubs [3], a situation similar to our study. Araújo (2015) [19] determined intake partition by sheep during the rainy season (April) on *Caatinga*-native pasture through micro-histological analyses of feces, and, according to intake level, for preferred and desirable species, the following species were observed: *Amaranthus blitum*, *Alternanthera tenella* Colla, *Alternanthera brasiliana* (L.) Kuntze, *Auxemma oncoalyx*, *Mimosa caesalpiniaefolia*, *Libidibia ferrea*, *Ipomoea* sp., *Sesuvium portulacastrum*, *Arachis* sp., *Centrosema* sp., *Stylosanthes humilis*, *Cyperus uniciualatus* Schrad. ex Ness, *Cynodon dactilon*, *Herissantia tiubae*, K.Schum. Brizicky, *Melochia corchorifolia* L., *Melochia pyramidata* L., *Wissadula rostrata*, and *Mimosa tenuiflora*, accounting for 52.6% of the key species that represent the sheep’s diet in this research.

The proportion of species in the diet selected by the animals has different proportions of pasture composition [6]. Variations in the dietary botanical composition of sheep are related to the eating strategies in native pasture areas, ability to select the diet in a heterogeneous pasture area, and, at the same time, with a large number of species, combined with the ability to select in a condition of adequate forage availability [26].

The intake obtained from the sum of the intake partition was on average 552 g DM, with adequate partition estimation verified by using LIPE^®^ and KL. However, it is worth highlighting that results were adequate and in line with other research findings when key species were used, adjusting them to the model through the double marker [18,27].

## 5. Conclusions

Based on the finding of this study, Zn and PG supplementation did not improve sheep nutrient intake when grazing *Caatinga*-native pasture in the rainy season. Moreover, *Caatinga*-native pasture biomass appears to have adequate protein and digestible organic matter levels during the early rainy season. Over this period; however, the advanced maturity of the plants and the reduced availability of pasture may result in intake variations by the animals. Furthermore, the low energy supply due to poor pasture quality between the months of April to June contributes to inefficient protein utilization and, consequently, reduced feed intake.

## Figures and Tables

**Table 1:** Herbaceous stratum availability, expressed as DM, and floristic composition of *Caatinga*-native pasture in the rainy season.

DM Availability, kg ha^−1^			Floristic Composition, g kg^−1^	
Legumes	Grasses	Total	Legumes	Grasses
1364	533	1897	719	281

**Table 2:** Frequency of main herbaceous species in *Caatinga*-native pasture during the rainy season.

Plant Species	Frequency	
	Absolute Number	Relative, %
*Arachis dardani* Krapov. and W.C. Greg.	23	3.69
*Oxalis corniculata* L.	92	14.7
*Hyptis suaveolens*	84	13.5
*Alternanthera tenella colla*	03	0.481
*Acalypha communis*	88	14.1
*Aspilia martii* Baker	20	3.21
*Centrosema Pascuorum* Mart. Ex Benth.	59	9.46
*Alternanthera brasiliana* (L.) Kuntze	41	6.57
*Stylosanthes humilis*	10	1.60
*Merremia aegyptia*	49	7.85
*Commelina diffusa*	46	7.37
*Digitaria Sanguinalis* (L.). Scop	54	8.65
*Wissadula rostrata*	55	8.81
Total	624	100

**Table 3:** Bromatological composition of *Caatinga*-native pasture during the rainy season.

Plant Species (Scientific Name)	Nutrients, g kg^−1^ DM									
	DM	OM	CP	EE	NDF	ADF	HCEL	CEL	ADL	LK
*Grasses*										
*Cyperus uniciualatus*	438	916	102	53.2	667	373	295	348	21.4	170
Native grass (*Cynodon* sp.)	420	867	87.1	58.3	625	332	292	281	44.8	156
*Digitaria Sanguinalis* (L.). Scop	282	935	97.0	55.4	696	351	345	271	258	166
*Legumes*										
*Delilia biflora* (L.) Kuntze	177	783	185	66.5	486	347	139	211	129	255
*Euphorbia heterophylla* L.	382	930	125	67.3	369	289	80.3	218	210	175
*Arachis dardani*	363	929	148	53.0	471	329	143	258	168	192
*Oxalis corniculata* L.	172	930	197	70.4	458	324	134	236	224	257
*Hyptis suaveolens*	144	890	175	86.0	544	368	177	176	219	410
*Sesuvium portulacastrum*	106	792	246	46.6	347	196	151	835	41.5	149
*Amaranthus blitum*	167	860	256	76.4	539	241	297	127	114	269
*Alternathera tenella Colla*	171	885	161	53.0	542	352	191	282	269	179
*Aspilia martii* Baker	251	896	184	74.0	501	359	143	230	206	268
*Centrosema Pascuorum*	220	930	223	66.1	482	352	130	260	242	271
*Alternanthera brasiliana*	135	842	213	46.5	488	270	218	187	76,6	176
*Stylosanthes humilis*	171	877	180	70.0	430	285	144	203	161	214
*Heliontropium sp.*	151	798	295	44.1	436	289	147	198	185	336
*Merremia aegyptia*	206	895	198	50.0	499	373	126	217	130	339
*Commelina difusa*	149	849	214	61.0	531	328	203	244	166	194
*Wissadula rostrata*	267	901	193	70.0	505	270	235	304	182	233
*Borreria verticillata*	387	904	132	48.9	428	347	81.0	291	67.0	198
*Shrub and arboreal*										
*Anadenathera colubrina*	598	931	96,0	49.0	229	157	71.4	202	131	395
*Caesalpinea pyramidalis*	394	935	166	80.0	383	230	153	208	92.0	183
*Zizyphus joazeiro*	472	934	177	44.8	609	368	241	207	160	325
*Libidibia férrea*	427	952	210	157	294	197	96.9	218	77.9	168
*Mimosa tenuiflora*	368	905	181	85.0	533	376	156	181	215	439
*Croton sonderianus*	297	920	171	84.0	496	348	148	194	169	412
*Combretum leprosum*	345	941	118	85.4	684	487	197	288	170	538
*Auxemma oncocalix*	366	878	201	54.0	674	479	195	226	234	362
*Mimosa caesalpinifolia*	361	938	191	115	642	403	238	193	212	505

DM = dry matter; OM = organic matter; CP = crude protein; EE = ether extract; NDF = neutral detergent fiber; ADF = acid detergent fiber; HCEL = hemicellulose; CEL = cellulose; ADL = acid detergent lignin (sa); KL = Kason lignin.

**Table 4:** Composition of ruminal extrusa samples^†^ collected from sheep on *Caatinga*-native pasture during the rainy season.

Variables	Periods				Concentrate ^β^
	March	April	May	June	
MS ^¥^, g kg^−1^	118	128	142	158	877
g kg^−1^ DM					
OM	819	810	798	819	913
CP	192	187	176	131	254
NDIN	2.99	2.87	3.03	3.01	3.04
NDIN, % of total nitrogen	98.3	96.3	108	145	74.6
Ether Extract	76.0	76.5	86.8	111	64.0
NDF	524	590	610	564	159
aNDFom-NDF ^‡^	437	496	504	478	113
ADF	430	476	487	473	103
Hemicellulose	94.1	114	123	91.5	56.1
Cellulose	208	250	261	243	45.6
Acid Detergent Lignin	35.4	45.4	52.5	37.8	11.3
Klason Lignin	40.7	50.4	65.4	52.8	17.8
Total tannins	0.64	8.14	8.33	14.8	-
IVDMD ^†^	537	408	424	441	954
IVOMD	468	333	353	359	939

^†^ Ruminal extrusa samples collected prior to rumen emptying after a one-hour grazing in thinning Caatinga area, IVDMD to according [16]; ^β^ Corn, soybean meal and limestone; ^¥^ Dry matter on a natural matter basis; ^‡^ aNDFom-NDF assayed with a heat stable amylase and expressed exclusive of residual ash NDF.

**Table 5:** Effects of zinc or propylene glycol supplementation on nutrient and fibrous fractions intake in sheep on *Caatinga*-native pasture.

Variables	Treatments ^‡^			Periods ^β^				SEM ^¥^	*p-*Value ^†^		
	CT	Zn	PG	Mar	Apr	May	Jun		T	P	T × P
Organic Matter											
g day^−1^	537	537	546	628 ^a^	551 ^b^	503 ^b^	502 ^b^	5.71	0.56	<0.01	0.57
g/kgLW^0.75^	58.1	55.3	56.0	68.0 ^a^	57.9 ^b^	52.0 ^c^	49.7 ^c^	0.21	0.49	<0.01	0.93
Crude Protein											
g day^−1^	75.5	74.7	71.7	101 ^a^	82.1 ^b^	62.6 ^c^	49.5 ^d^	1.03	0.33	<0.01	0.58
g/kgLW^0.75^	7.62	7.13	7.38	11.0 ^a^	8.56 ^b^	6.44 ^c^	4.88 ^d^	0.06	0.41	<0.01	0.82
Neutral Detergent Fiber											
g day^−1^	246	234	243	290 ^a^	239 ^b^	231 ^b^	203 ^c^	3.17	0.33	<0.01	0.52
g/kgLW^0.75^	25.5	23.9	24.7	31.4 ^a^	25.0 ^b^	23.8 ^b^	20.0 ^c^	0.13	0.39	<0.01	0.82
Acid Detergent Fiber											
g day^−1^	207	197	205	251 ^a^	205 ^b^	181 ^c^	173 ^cd^	2.68	0.35	<0.01	0.53
g/kgLW^0.75^	21.4	20.1	20.8	27.1 ^a^	21.4 ^b^	18.7 ^c^	17.1 ^c^	0.12	0.40	<0.01	0.82
Cellulose											
g day^−1^	106	101	105	130 ^a^	107 ^b^	97.7 ^c^	84.3 ^d^	1.39	0.35	<0.01	0.53
g/kgLW^0.75^	11.0	10.3	10.7	14.0 ^a^	11.1 ^b^	9.95 ^b^	8.32 ^c^	0.08	0.40	<0.01	0.82

^a–d^ Means in the same line followed by different letters are different by the Tukey-Kramer test (*p* < 0.05). ^‡^ CT = control; Zn = ZnSO_4_.7H_2_O addition for supply of 300 mg Zn day^−1^ in the salt; PG = addition of 2.5 mL/kgLW^0.75^ animal^−1^ day^−1^ of propylene glycol mixed with the concentrate. ^β^ Mar = March; Apr = April; May = May; Jun = June; ^¥^ SEM = standard error of the mean; ^†^ T = treatment; P = period; T × P = interaction between treatments and periods.

**Table 6:** Effects of zinc sulfate or propylene glycol supplementation on nutrient digestibility in sheep on *Caatinga*-native pasture.

Variables ^£^	Treatments ^‡^			Periods ^β^				SEM ^¥^	*p-*Value ^†^		
	CT	Zn	PG	Mar	Apr	May	Jun		T	P	T × P
*Digestibility Coefficient, %*											
OMD^£^	54.7 ^b^	53.5 ^b^	56.2 ^a^	59.8 ^a^	54.5 ^b^	53.8 ^b^	51.1 ^c^	0.35	0.01	<0.01	0.83
CPD	36.0	36.1	40.2	52.6 ^a^	40.8 ^b^	33.7 ^c^	22.6 ^d^	0.87	0.09	<0.01	0.87
NDFD	47.9	44.9	48.0	56.9 ^a^	47.2 ^b^	49.2 ^b^	34.3 ^c^	0.58	0.05	<0.01	0.73
ADFD	50.0 ^a^	46.6 ^b^	50.3 ^a^	60.9 ^a^	48.3 ^b^	48.0 ^b^	38.7 ^c^	0.55	0.01	<0.01	0.46
CELD	41.1	39.8	40.8	53.8 ^a^	39.3 ^b^	40.3 ^b^	28.8 ^c^	0.79	0.80	<0.01	0.57

^a–d^ Means in the same line followed by different letters are different by the Tukey-Kramer test (*p* < 0.05). ^‡^ CT = control; Zn = ZnSO_4_.7H_2_O addition for supply of 300 mg Zn day^−1^ in the salt; PG = addition of 2.5 mL/ g/kgLW^0.75^ animal^−1^ day^−1^ of propylene glycol mixed with the concentrate. ^β^ Mar = March Apr = April; May = May; Jun = June; ^£^ OMD, CPD, NDFD, ADFD, CELD = OM, CP, NDF, ADF, and cellulose digestibility. ^¥^ SEM = Standard Error of the Mean; ^†^ T = treatment; P = period; T × P = interaction between treatments and period.

**Table 7:** Effects of zinc sulfate or propylene glycol supplementation on the pasture selection in sheep on *Caatinga*-native pasture.

Intake Partition, g day^−1^ (on a DM Basis)	Treatments			Periods ^β^				SEM ^¥^	*p-*Value ^†^		
	CT	Zn	PG	Mar	Apr	May	Jun		T	P	T × P
*Grasses*											
*Cyperus uniciualatus* Schrad. ex Ness	22.8	22.2	22.6	22.8 ^ab^	23.4 ^a^	21.6 ^b^	22.2 ^ab^	0.19	0.46	0.0	0.32
Gramínea Nativa (*Cynodon* sp.)	19.9	19.4	19.8	19.9 ^ab^	20.4 ^a^	18.9 ^b^	19.5 ^ab^	0.17	0.48	0.01	0.31
*Digitaria Sanguinalis* (L.). Scop	21.2	20.7	21.1	21.3 ^ab^	21.8 ^a^	20.1 ^b^	20.8 ^ab^	0.18	0.46	<0.01	0.32
*Herbaceous Legumes*											
*Alternanthera brasiliana* Mart.	22.0	21.4	21.9	22.1 ^ab^	22.6 ^a^	20.9 ^b^	21.5 ^ab^	0.19	0.48	<0.01	0.32
*Alternathera tenella* Colla	24.5	23.9	24.3	24.6 ^ab^	25.2 ^a^	23.2 ^b^	23.9 ^ab^	0.21	0.45	<0.01	0.32
*Amaranthus blitum*	38.6	37.6	38.3	38.7 ^ab^	40.0 ^a^	36.7 ^b^	37.3 ^b^	0.33	0.47	<0.01	0.32
*Arachis dardani*	24.2	23.6	24.1	24.3 ^ab^	25.0 ^a^	23.0 ^b^	23.6 ^ab^	0.21	0.46	<0.01	0.32
*Aspilia martii Baker*	26.8	26.8	27.3	27.6 ^ab^	28.4 ^a^	26.1 ^b^	26.8 ^ab^	0.24	0.46	<0.01	0.33
*Borreria verticillat*)	25.8	25.1	25.6	25.8 ^ab^	26.6 ^a^	24.5 ^b^	25.1 ^ab^	0.22	0.47	<0.01	0.31
*Centrosema* sp.	38.2	37.2	38.0	38.3 ^ab^	39.6 ^a^	36.2 ^b^	37.0 ^b^	0.33	0.46	<0.01	0.31
*Commelina difusa*	31.6	30.8	31.4	31.6 ^ab^	32.7 ^a^	30.0 ^b^	30.7 ^b^	0.27	0.46	<0.01	0.32
*Delilia biflora* (L.) Kuntze	34.7	33.8	34.5	34.8 ^ab^	36.0 ^a^	33.0 ^b^	33.6 ^b^	0.30	0.47	<0.01	0.31
*Oxalis corniculata* L.	34.6	33.7	34.4	34.7 ^ab^	35.9 ^a^	32.9 ^b^	33.5 ^b^	0.29	0.47	<0.01	0.31
*Sesuvium portulacastrum*	19.7	19.2	19.5	19.7 ^ab^	20.2 ^a^	18.6 ^b^	19.3 ^ab^	0.17	0.46	<0.01	0.33
*Stylosanthes humilis*	25.9	25.3	25.8	26.0 ^ab^	26.7 ^a^	24.6 ^b^	25.3 ^ab^	0.22	0.48	<0.01	0.31
*Wissadula rostrata*	38.1	37.1	37.8	38.1 ^ab^	39.5 ^a^	36.2 ^b^	36.8 ^b^	0.33	0.46	<0.01	0.31
*Arboreal Legumes*											
*Auxemma oncocalix*	49.6	48.3	49.3	49.7 ^ab^	51.6 ^a^	47.2 ^b^	47.8 ^b^	0.42	0.45	<0.01	0.32
*Mimosa caesalpinifolia*	24.1	23.4	23.9	24.1 ^ab^	24.8 ^a^	22.8 ^b^	23.5 ^ab^	0.21	0.46	<0.01	0.33
*Zizyphus joazeiro*	35.5	34.6	35.5	46.3 ^a^	33.1 ^b^	30.4 ^c^	31.1 ^bc^	0.32	0.48	<0.01	0.32
*Total Intake*	558	544	555	570 ^a^	573 ^a^	526 ^b^	539 ^a^	4.79	0.46	<0.01	0.31

^a–c^ Means in the same line followed by different letters are different by the Tukey-Kramer test (*p* < 0.05). ^‡^ CT = control; Zn = ZnSO_4_.7H_2_O addition for supply of 300 mg Zn day^−1^ in the salt; PG = addition of 2.5 mL/ g/kgLW^0.75^ animal^−1^ day^−1^ of propylene glycol mixed with the concentrate. ^β^ Mar = March; Apr = April; May = May; Jun = June; ^¥^ SEM = standard error of the mean; ^†^ T = treatment; P = period; T × P = interaction between treatments and periods.
